# Hemichorea after hyperglycemia correction: A case report and a short review of hyperglycemia-related hemichorea at the euglycemic state

**DOI:** 10.1097/MD.0000000000010076

**Published:** 2018-03-09

**Authors:** Hsiao-Shan Cho, Chien-Tai Hong, Lung Chan

**Affiliations:** a Department of Neurology, Shuang Ho Hospital, New Taipei City; b Department of Neurology, School of Medicine, College of Medicine, Taipei Medical University, Taipei, Taiwan.

**Keywords:** basal ganglia, chorea, euglycemic state, hyperglycemia, ketone body

## Abstract

**Rationale:**

Hyperglycemic hemichorea tends to affect elderly patients with type 2 diabetes, women, and the Asian population. The onset of involuntary movement typically occurs at the hyperglycemic state and subsides at the euglycemic state. In this report, we present an unusual case that developed delayed-onset hemichorea after hyperglycemia correction.

**Patient concerns:**

A 70-year-old man was admitted to neurology ward with symptoms of subacute dizziness. Hyperglycemia and high level ketone body was incidentally noted. Hemichorea occurred in his left limbs 2 days after hyperglycemia correction.

**Diagnoses:**

Patient remained conscious, and no other focal neurological deficits were noted while hemichorea occurred. Blood test revealed no contributory cause. Brain magnetic resonance imaging revealed no lesions in the putamen or subthalamus. A diagnosis of probable hyperglycemia-related hemichorea was made.

**Interventions:**

Haloperidol (2 mg, 3 times per day) was prescribed.

**Outcomes:**

Hemichorea improved gradually before discharge and resolved 4 months later.

**Lessons:**

Differential diagnosis of hemichorea should include delayed-onset hemichorea after hyperglycemia correction.

## 1 Introduction

Hemichorea is an uncommon but remarkable neurological disorder associated with hyperglycemia. Elderly patients with type 2 diabetes, women, and the Asian population have a higher risk of hyperglycemic hemichorea.^[1]^ The onset of hemichorea typically occurs at the hyperglycemic state and subsides after hyperglycemia correction. Here, we report a rare case of acute hemichorea that occurred after hyperglycemia correction.

## 2 Case report

**Figure 1:**
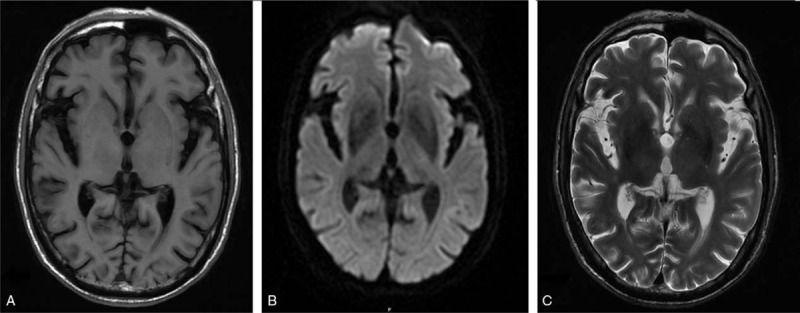
Brain magnetic resonance imaging showed no infarction or hyperintensity lesion in the right basal ganglion (A) T1-weighted brain magnetic resonance imaging (B) diffusion-weighted imaging (C) T2-weighted brain magnetic resonance imaging.

A 70-year-old male patient presented to the emergency department with a 2-day history of dizziness. The patient denied diplopia, blurred vision, headache, focal numbness, and weakness. Neurological examination showed no focal neurological signs. Laboratory data revealed elevated levels of blood sugar (415 mg/dL), glycated hemoglobin (19%), osmolality (301 mOsm/kg H_2_O), and blood ketone bodies (5.9 mmol/L). Renal functions, hepatic functions, and blood gas were within the normal range. Brain computed tomography performed at the emergency department revealed negative result. Subsequently, the patient was admitted, and oral hypoglycemic agents including Metformin (1000 mg/d) and Glimepiride (2 mg/d) were prescribed; intermittent subcutaneous insulin was administered if the blood sugar level increased >200 mg/dL. After 2 days, acute hemichorea was observed in his left limbs; the blood sugar level was 168 mg/dL. The blood sugar level did not increase during these 2 days. He remained conscious, and no other focal neurological deficits were noted. Other laboratory test results were within the normal range, including the blood cell count, renal function, liver function, thyroid function, vitamin B12 level, autoimmune profile, human immunodeficiency virus antibody titer, and rapid plasma reagin. Brain magnetic resonance imaging (MRI) performed after hemichorea onset showed no infarction or hyperintensity lesion in the right basal ganglion and subthalamus (Fig. 1). Hyperglycemia-related hemichorea was diagnosed after excluding other metabolic encephalopathy and focal cerebral lesions. Haloperidol (2 mg, 3 times per day) was prescribed, and hemichorea improved gradually and resolved after 4 months after discharge; the blood sugar level was 138 mg/dL; glycated hemoglobin was 7.1%.

## 3 Discussion

Generally, the blood sugar level is extremely high during hemichorea onset. However, in this case report, we present an unusual disease course, in which the involuntary movement occurred after hyperglycemia correction. In the literature, 2 cases of delayed-onset hemichorea after hyperglycemia correction have been reported. Bizet et al^[2]^ presented a case report of a 66-year-old woman with hyperosmolar hyperglycemic nonketotic syndrome. Three months after admission, she developed chorea for 1 week at the euglycemic state (blood sugar level: 84 mg/dL). However, because of the prolonged interval between hyperglycemia and onset of hemichorea, the relationship between them remains debatable. Taboada et al^[3]^ reported a case of an elderly female patient with transient ischemic attack and hyperglycemia (blood sugar level: 700 mg/dL) at the initial presentation. Hemichorea occurred 1 week after partial hyperglycemia correction (blood sugar level: 486 mg/dL). However, the blood sugar level did not return to its normal range during hemichorea onset.

The most commonly reported neurological image findings among the hyperglycemic hemichorea cases were lesions in the putamen contralateral to the affected limbs.^[1]^ However, the association between neuroimaging findings and symptoms varied. Some cases had no typical neuroimaging lesions in the basal ganglion, whereas others had bilateral neurological lesions associated with hemichorea.^[3,4]^ The pathological changes in putaminal lesions remain unclear. Advanced neuroimaging studies, such as magnetic resonance spectroscopy (MRS), single-photon emission computed tomography (SPECT), and 2-[18F] fluoro-2-deoxy-D-glucose-positron emission tomography (FDG-PET), have provided some possible explanations. MRS revealed relatively low N-acetylaspartate to creatine ratio, high choline to creatine ratio, and lactate peak, indicating neuronal damage, gliosis, and acute or chronic ischemic changes, respectively.^[5]^ Furthermore, SPECT suggested hypoperfusion in the striatum,^[6]^ and FDG-PET demonstrated reduced rates of cerebral glucose metabolism in the corresponding areas with abnormalities on T1-weighted MRI.^[7]^

The molecular pathogenesis underlying hyperglycemic hemichorea remains unclear, and 2 major hypotheses have been proposed. First, γ-aminobutyric acid (GABA) was used as an alternate energy substrate during hyperglycemic crisis. GABA depletion leads to thalamic disinhibition and hyperkinesia.^[3,8]^ Second, vasculopathy was hypothesized to be a possible mechanism. Long-term hyperglycemia causes hyperviscosity of blood, which leads to latent ischemia of the striatum and subsequent dyskinesia.^[3]^ Although this hypothesis was not confirmed in most cases with unilateral lesions, it was partially supported by a striatal biopsy after hyperglycemia-related hemichorea, which showed neuron loss, gliosis, and reactive astrocytosis.^[9]^ Ketone bodies may play a role in hyperglycemia-related hemichorea. The absence of insulin leads to hyperglycemia and the release of free fatty acids from the adipose tissue, which are metabolized to ketone bodies (acetoacetateand β-hydroxybutyrate). Acetoacetatecan can be used as a GABA substitute to temporarily compensate for the hyperglycemia-induced GABA depletion.^[10]^ Thus, this finding reflects the rarity of hemichorea in patients with diabetic ketoacidosis.

Our patient demonstrated a remissive course of involuntary movement and in absence of putaminal abnormalities on MRI, which support the hypothesis that hyperglycemia self plays a major pathogenetic role. Delayed chorea may due to temporarily compensate for the GABA depletion by high level ketone bodies initially. However, our hypothesis has some limitations. First, ketone bodies level was not mentioned in the previous case reports for delayed hyperglycemia-related chorea.^[2,3]^ We are the first one who mentioned ketone bodies play a role in this condition, which stress the need for further studies to prove this hypothesis. Second, in a small review for hemichorea in ketotic hyperglycemia, no patient presented with delayed hemichorea after hyperglycemia correction.^[10]^

## 4 Conclusion

Although a rare possibility, differential diagnosis of hemichorea should include delayed-onset hemichorea after hyperglycemia correction. We hypothesize that hyperglycemia-induced ketone bodies play a crucial role in delayed-onset hemichorea; however, the underlying molecular pathogenesis remains unclear.

## Acknowledgments

The authors acknowledge Wallace Academic Editing for editing this manuscript.
